# Application of the backstepping method to the prediction of increase or decrease of infected population

**DOI:** 10.1186/s12976-016-0041-6

**Published:** 2016-05-10

**Authors:** Toshikazu Kuniya, Hideki Sano

**Affiliations:** Department of Applied Mathematics, Graduate School of System Informatics, Kobe University, 1-1 Rokkodai-cho, Nada-ku, Kobe, 657-8501 Japan

**Keywords:** Backstepping method, Age structure, Prediction, Influenza, ARIMA

## Abstract

**Background:**

In mathematical epidemiology, age-structured epidemic models have usually been formulated as the boundary-value problems of the partial differential equations. On the other hand, in engineering, the backstepping method has recently been developed and widely studied by many authors.

**Methods:**

Using the backstepping method, we obtained a boundary feedback control which plays the role of the threshold criteria for the prediction of increase or decrease of newly infected population. Under an assumption that the period of infectiousness is same for all infected individuals (that is, the recovery rate is given by the Dirac delta function multiplied by a sufficiently large positive constant), the prediction method is simplified to the comparison of the numbers of reported cases at the current and previous time steps.

**Results:**

Our prediction method was applied to the reported cases per sentinel of influenza in Japan from 2006 to 2015 and its accuracy was 0.81 (404 correct predictions to the total 500 predictions). It was higher than that of the ARIMA models with different orders of the autoregressive part, differencing and moving-average process. In addition, a proposed method for the estimation of the number of reported cases, which is consistent with our prediction method, was better than that of the best-fitted ARIMA model *ARIMA*(1,1,0) in the sense of mean square error.

**Conclusions:**

Our prediction method based on the backstepping method can be simplified to the comparison of the numbers of reported cases of the current and previous time steps. In spite of its simplicity, it can provide a good prediction for the spread of influenza in Japan.

## Background

The spreading mechanism of infectious diseases in population has been studied in terms of mathematical modeling since the pioneering works by Kermack and McKendrick [1, 2] (see Hethcote [3] for a detailed review). In the eradication or reduction of diseases, the concept of control would play an important role (see, for instance, Anderson and May [4] and Smith et al. [5]). To design the appropriate control strategies, the prediction of epidemic size is essentially important. In recent years, Ferguson et al. [6], Hyder et al. [7] and Riley et al. [8] studied influenza and other diseases from this perspective. On the other hand, the qualitative analysis of mathematical models is also important for understanding the effect of control. Alexander et al. [9] and Mills et al. [10] published their study from this perspective.

For a long time, age-structured epidemic models have been studied by many authors (see, for instance, Iannelli [11], Inaba [12], Inaba and Nishiura [13] and Tudor [14]). Mathematically, these models can be regarded as the boundary-value problems of partial differential equations. On the other hand, in engineering, the backstepping method has recently been developed by Smyshlyaev and Krstic [15] to obtain the boundary feedback control for stabilizing the systems of partial differential equations and has widely been studied by many authors (see, for instance, Susto and Krstic [16] and Baccoli et al. [17]). The aim of this study is to make use of the backstepping method for epidemiological considerations. Specifically, we will develop a new method for the prediction of increase or decrease of infected population.

In the classical theory of the basic reproduction number ${\mathcal {R}}_{0}$ (see, for instance, Diekmann et al. [18] and van den Driessche and Watmough [19]), the number of newly infected individuals produced by a typical infected individual invading into a fully susceptible population is characterized as the threshold value. That is, if ${\mathcal {R}}_{0} > 1$, then the epidemic size will increase and if ${\mathcal {R}}_{0} < 1$, then it will decrease. In our method, a threshold criteria *U*(*t*) will be calculated for each time step *t* as a boundary feedback control. It will be shown that whether the newly infected population *I*(*t*,0) at each time step *t* will exceed *U*(*t*) or not determines the increase or decrease of infected population at the next time step *t*+1. That is, if *I*(*t*,0)>*U*(*t*) (*I*(*t*,0)<*U*(*t*)) at the current time step *t*, then the infected population will increase (decrease) at the next time step *t*+1. Thus, we can predict the increase or decrease of infected population by comparing *I*(*t*,0) and *U*(*t*) for each time step.

## Methods

### The model formulation

The meaning of each symbol in our mathematical model is as follows.

- *t*≥0: the chronological time;
- *a*∈ [ 0,*a*_*†*_]: the class age (that is, the time elapsed since the infection) of infected individuals;
- *a*_*†*_>0: the maximum period of infectiousness;
- *I*(*t*,*a*): the infected population of class age *a* at time *t*;
- $\gamma (a) \in L^{1}_{+}(0,a_{\dagger })$: the per capita recovery rate of infected individuals of class age *a*.

Under this setting, the class age-structured epidemic model in this paper is formulated as follows.

1 $$ \left(\frac{\partial}{\partial t} + \frac{\partial}{\partial a}\right) I(t,a) = - \gamma (a) I(t,a), \quad t>0, \ \ 0 < a < a_{\dagger}.  $$

Note that the boundary condition for *a*=0 is not considered in (1) unlike usual class age-structured epidemic models. In fact, in this paper, we analytically derive the boundary feedback control *U*(*t*) by using the backstepping method and compare it with *I*(*t*,0) which will be obtained from the real data.

### The backstepping method

The aim of this section is to derive the boundary feedback control *U*(*t*). Let us consider the following integral transformation.

2 $$ w(t,a) := I(t,a) - \int_{a}^{a_{\dagger}} k(a,\sigma) I(t, \sigma) \mathrm{d}\sigma.  $$

Now we seek the integral kernel *k*(*a*,*σ*) such that *w*(*t*,*a*) becomes the solution of the following target system.

3 $$ \left\{ \begin{array}{ll} \vspace{.5em} \left(\frac{\partial}{\partial t} + \frac{\partial}{\partial a}\right) w(t,a) = - \gamma (a) w(t,a) + \psi(a) w(t,a_{\dagger}), & t > 0, \ \ 0 < a < a_{\dagger}, \\ w(t,0) = 0, & t > 0, \end{array} \right.  $$

where *ψ*(*a*) is a positive continuous function on [ 0,*a*_*†*_] which is chosen such that the operator describing the target system has the spectrum consisting of stable eigenvalues and zero eigenvalue, that is, *w*(*t*,*a*) converges to a nontrivial equilibrium *w*^∗^(*a*) as *t*→+*∞*. Therefore, we see that if the integral equation

4 $$ w^{*}(a) = I(t,a) - \int_{a}^{a_{\dagger}} k(a,\sigma) I(t,\sigma) \mathrm{d}\sigma  $$

has an equilibrium *I*^∗^(*a*), then *I*(*t*,*a*) converges to *I*^∗^(*a*) as *t*→+*∞*. Hence, in what follows, we will determine the integral kernel *k*(*a*,*σ*) such that (2) satisfies (3) and further (4) has the equilibrium *I*^∗^(*a*).

Differentiating (2) with respect to *t* and using (1), we have

5 $$\begin{array}{@{}rcl@{}} &&\quad \frac{\partial}{\partial t} w(t,a)  \\ && = \frac{\partial}{\partial t} I(t,a) - \int_{a}^{a_{\dagger}} k(a,\sigma) \frac{\partial}{\partial t} I(t,\sigma) \mathrm{d}\sigma  \\ &&= \frac{\partial}{\partial t} I(t,a) - \int_{a}^{a_{\dagger}} k(a,\sigma) \left[ -\frac{\partial}{\partial \sigma} I(t,\sigma) -\gamma(\sigma) I(t,\sigma) \right] \mathrm{d}\sigma  \\ && = \frac{\partial}{\partial t} I(t,a) - k(a,a) I(t,a) + k(a,a_{\dagger}) I(t,a_{\dagger})  \\ &&\quad - \int_{a}^{a_{\dagger}} \left[ \frac{\partial}{\partial \sigma} k(a,\sigma) -\gamma(\sigma) k(a,\sigma) \right] I(t,\sigma) \mathrm{d}\sigma.  \end{array} $$

On the other hand, differentiating (2) with respect to *a*, we have

6 $$ \frac{\partial}{\partial a} w(t,a) = \frac{\partial}{\partial a} I(t,a) + k(a,a) I(t,a) - \int_{a}^{a_{\dagger}} \frac{\partial}{\partial a} k(a,\sigma) I(t,\sigma) \mathrm{d}\sigma.  $$

Adding both sides of the Eqs. (5) and (6) and using (1), we have

$$\begin{array}{@{}rcl@{}} \left(\frac{\partial}{\partial t} + \frac{\partial}{\partial a} \right) w(t,a) &=& \left(\frac{\partial}{\partial t} + \frac{\partial}{\partial a} \right) I(t,a) + k(a,a_{\dagger}) I(t,a_{\dagger})  \\ && -\int_{a}^{a_{\dagger}} \left[ \left(\frac{\partial}{\partial a} + \frac{\partial}{\partial \sigma} \right) k(a,\sigma) -\gamma(\sigma) k(a,\sigma) \right] I(t,\sigma) \mathrm{d}\sigma  \\ &=& -\gamma (a) I(t,a) + k(a,a_{\dagger}) I(t,a_{\dagger})  \\ && -\int_{a}^{a_{\dagger}} \left[ \left(\frac{\partial}{\partial a} + \frac{\partial}{\partial \sigma} \right) k(a,\sigma) -\gamma(\sigma) k(a,\sigma) \right] I(t,\sigma) \mathrm{d}\sigma  \\ &=& -\gamma (a) w(t,a) + k(a,a_{\dagger}) w(t,a_{\dagger})  \\ && -\int_{a}^{a_{\dagger}} \left[ \left(\frac{\partial}{\partial a} + \frac{\partial}{\partial \sigma} \right) k(a,\sigma) + \left(\gamma(a) -\gamma(\sigma) \right) k(a,\sigma) \right] I(t,\sigma) \mathrm{d}\sigma.  \end{array} $$

Comparing this equation with (3), we see that *k*(*a*,*σ*) should satisfy the following equation.

7 $$ \left\{ \begin{array}{ll} \vspace{.5em} \left(\frac{\partial}{\partial a} + \frac{\partial}{\partial \sigma} \right) k(a,\sigma) + \left(\gamma(a) -\gamma(\sigma) \right) k(a,\sigma) = 0, & a \in \left(0, a_{\dagger} \right), \ \sigma \in \left(a, a_{\dagger} \right), \\ k(a,a_{\dagger}) = \psi(a), & a \in \left[0, a_{\dagger} \right]. \end{array} \right.  $$

Since the operator describing the target system (3) is required to have zero eigenvalue, *ψ*(*σ*) should satisfy

8 $$ \int_{0}^{a_{\dagger}} \psi (\sigma) \mathrm{e}^{-\int_{\sigma}^{a_{\dagger}} \gamma(\rho) \mathrm{d}\rho} \mathrm{d}\sigma = 1.  $$

Note that the Eq. (8) corresponds to the situation where the basic reproduction number ${\mathcal {R}}_{0}$ is just equal to one (see, for instance, Diekmann et al. [18]). By integrating (7) along the characteristic line *σ*−*a*=const., we obtain

9 $$ k(a,\sigma) = \psi(a_{\dagger}+a-\sigma) \mathrm{e}^{\int_{0}^{a_{\dagger}-\sigma} \left(\gamma(a_{\dagger}+a-\sigma-\rho) -\gamma(a_{\dagger}-\rho) \right) \mathrm{d}\rho}, \quad \sigma \in (a, a_{\dagger}).   $$

Since the kernel *k*(*a*,*σ*) is bounded on the domain 0≤*a*≤*σ*≤*a*_*†*_ and *w*^∗^(*a*) is continuous on [ 0,*a*_*†*_], the integral Eq. (4) has an equilibrium *I*^∗^(*a*) and the solution *I*(*t*,*a*) of (1) converges to *I*^∗^(*a*) as *t*→+*∞* under the boundary condition

$$\begin{array}{@{}rcl@{}} I(t,0) &=& \int_{0}^{a_{\dagger}} k(0,\sigma) I(t,\sigma) \mathrm{d}\sigma \\ &=& \int_{0}^{a_{\dagger}} \psi(a_{\dagger}-\sigma) \mathrm{e}^{\int_{0}^{a_{\dagger}-\sigma} \left(\gamma(a_{\dagger}-\sigma-\rho) -\gamma(a_{\dagger}-\rho) \right) \mathrm{d}\rho} I(t,\sigma) \mathrm{d}\sigma. \end{array} $$

Thus, the boundary feedback control *U*(*t*) is given by

10 $$ U(t) := \int_{0}^{a_{\dagger}} \psi(a_{\dagger}-\sigma) \mathrm{e}^{\int_{0}^{a_{\dagger}-\sigma} \left(\gamma(a_{\dagger}-\sigma-\rho) -\gamma(a_{\dagger}-\rho) \right) \mathrm{d}\rho} I(t,\sigma) \mathrm{d}\sigma  $$

and if *I*(*t*,0)>*U*(*t*), then the infected population *I*(*t*,*a*) will increase and diverge, while if *I*(*t*,0)<*U*(*t*), then the infected population *I*(*t*,*a*) will decrease and converge to 0.

#### Remark on mathematical well-posedness

If we consider the initial condition *I*_0_(*a*):=*I*(0,*a*), it should satisfy the following condition to let (1) have a differentiable solution *I*(*t*,*a*):

11 $$\begin{array}{@{}rcl@{}} &&I_{0} (\cdot) \in H^{1} (0,a_{\dagger}),  \\ &&I_{0}(0) = \int_{0}^{a_{\dagger}} \psi(a_{\dagger}-\sigma) \mathrm{e}^{\int_{0}^{a_{\dagger}-\sigma} \left(\gamma(a_{\dagger}-\sigma-\rho) -\gamma(a_{\dagger}-\rho) \right) \mathrm{d}\rho} I_{0}(\sigma) \mathrm{d}\sigma.  \end{array} $$

In fact, if the first two conditions are satisfied, we can show the existence of the differentiable solution *I*(*t*,*a*) by constructing a *C*_0_-semigroup generated by the differentiation operator −d/d*a*−*γ*(*a*) with the domain $\{I(\cdot) \in H^{1} (0,a_{\dagger }) ; I(0) = \int _{0}^{a_{\dagger }} \psi (a_{\dagger }-\sigma) \mathrm {e}^{\int _{0}^{a_{\dagger }-\sigma } \left (\gamma (a_{\dagger }-\sigma -\rho) -\gamma (a_{\dagger }-\rho) \right) \mathrm {d}\rho } I(\sigma) \mathrm {d}\sigma \}$. Since the aim of this paper is to develop a prediction method, we do not require the observed data to satisfy the condition (11). However, we remark that this mathematical rigorousness is essentially important from the analytical point of view.

### The prediction method

From the above discussion, we obtain the following prediction guideline.

12 $$ \begin{array}{l} \text{If}~\ I(t,0) > U(t), \ \mathrm{then \ the \ infected \ population} \ I(t,a) \ \mathrm{will \ increase.} \\ \text{If} ~\ I(t,0) < U(t), \ \mathrm{then \ the \ infected \ population} \ I(t,a) \ \mathrm{will \ decrease.} \end{array}  $$

**Fig. 1:**
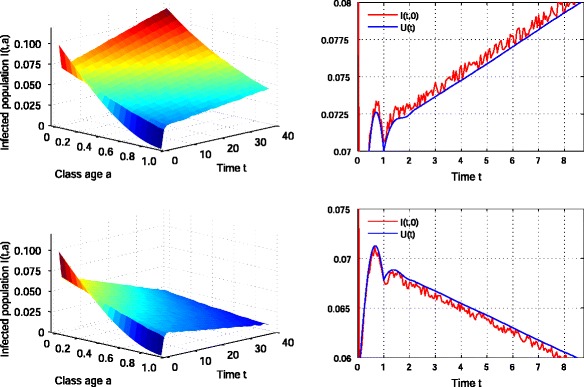
The decrease and increase of the infected population depending on the newly infected population *I*(*t*,0). Let *a*_*†*_=1, *γ*(*a*)≡1 and *X*∈(0,1) be a uniform random variable. For *I*(*t*,0)=*U*(*t*)+0.001*X*(>*U*(*t*)), the infected population *I*(*t*,*a*) increases and will diverge (*upper two figures*). For *I*(*t*,0)= max(*U*(*t*)−0.001*X*,0)(<*U*(*t*)), the infected population *I*(*t*,*a*) decreases and will converge to 0 (*lower two figures*)

In fact, we obtain Fig. 1 for some artificial parameters, which is an example for verifying the validity of (12).

In what follows, for simplicity, we assume that *ψ*(*a*)≡*ψ* and the period of infectiousness is same for all infected individuals. To represent this situation, we assume that the recovery rate is given by the Dirac delta function multiplied by a sufficiently large positive constant *M*≫0: *γ*(*a*)=*Mδ*(*a*−*a*_*†*_). Here, a model function for the Dirac delta function can be given by the isosceles triangle with height *m*>0 and base *a*_*†*_−2/*m*<*a*<*a*_*†*_ (which approaches to *δ*(*a*−*a*_*†*_) as *m*→+*∞*). In this case, the survival rate at which an infected individual of class age *a* still has infectiousness is given by

$$\Gamma (a) := \mathrm{e}^{-{\int_{0}^{a}} \gamma (\sigma) \mathrm{d}\sigma} = \mathrm{e}^{-M {\int_{0}^{a}} \delta (\sigma-a_{\dagger}) \mathrm{d}\sigma} = \left\{ \begin{array}{ll} 1, & a < a_{\dagger}, \\ \mathrm{e}^{-M} \left(\approx 0 \right), & a = a_{\dagger}. \end{array} \right. $$

From (8), we have

$$\psi \int_{0}^{a_{\dagger}} \mathrm{e}^{-M} \mathrm{d}\sigma = \psi \mathrm{e}^{-M} a_{\dagger} = 1 $$

and hence, *ψ*=e^*M*^/*a*_*†*_. Then, from (10), the boundary feedback control *U*(*t*) is given by

$$\begin{array}{@{}rcl@{}} U(t) &=& \frac{\mathrm{e}^{M}}{a_{\dagger}} \int_{0}^{a_{\dagger}} \mathrm{e}^{M\int_{0}^{a_{\dagger} -\sigma} \left(\delta(-\sigma-\rho) - \delta(-\rho) \right) \mathrm{d}\rho} I(t,\sigma) \mathrm{d}\sigma \\ &=& \frac{\mathrm{e}^{M}}{a_{\dagger}} \int_{0}^{a_{\dagger}} \mathrm{e}^{-M} I(t,\sigma) \mathrm{d}\sigma \ = \ \frac{1}{a_{\dagger}} \int_{0}^{a_{\dagger}} I(t,\sigma) \mathrm{d}\sigma \\ &=& \frac{1}{a_{\dagger}} \int_{0}^{a_{\dagger}} I(t-\sigma, 0) \mathrm{e}^{-M\int_{0}^{\sigma} \delta (\rho -a_{\dagger}) \mathrm{d}\rho}\mathrm{d}\sigma \ = \ \frac{1}{a_{\dagger}} \int_{0}^{a_{\dagger}} I(t-\sigma, 0) \mathrm{d}\sigma. \end{array} $$

Let us consider the discrete-time series and let *a*_*†*_=1. Then, by using the rectangle approximation at *σ*=*a*_*†*_=1, the above equality can be represented as follows.

13 $$ U(t) \approx I(t-1, 0).  $$

Thus, from (12), we can predict that if

$$\begin{array}{l} \hspace{0em} \left(\mathrm{the \ number \ of \ reported \ cases \ at \ the \ current \ time \ step} \ t \right) \\ \hspace{0em} > \ \left(\mathrm{the \ number \ of \ reported \ cases \ at \ the \ previous \ time \ step} \ t-1 \right) \end{array} $$

then the number of reported cases at the next time step *t*+1 will increase, and if

$$\begin{array}{l} \hspace{0em} \left(\mathrm{the \ number \ of \ reported \ cases \ at \ the \ current \ time \ step} \ t \right) \\ \hspace{0em} < \ \left(\mathrm{the \ number \ of \ reported \ cases \ at \ the \ previous \ time \ step} \ t-1 \right) \end{array} $$

then the number of reported cases at the next time step *t*+1 will decrease. Although this prediction method is very simple, its accuracy can be high for the real data of the spread of influenza in Japan (see the next section).

## Results and discussion

### Prediction of increase or decrease of infected population for influenza in Japan

In this section, we apply our prediction method proposed above to the real data of reported cases of influenza in Japan from 2006 to 2015. The data is available in the website of the National Institute of Infectious Diseases, Japan (see [20]). Let the unit time step be a week.

First, in Table 1, we exhibit the result of our prediction from the week 1 to week 12 in 2015.

**Table 1:** The actual number of reported cases per sentinel and prediction results for influenza in Japan from week 1 to week 12 in 2015

Week (*t*)	Number of reported	*U*(*t*)	Prediction	Result
	cases per sentinel			
1	21.46	–	–	–
2	33.28	21.46	Increase	Correct
3	37	33.28	Increase	Correct
4	39.42	37	Increase	Incorrect
5	29.11	39.42	Decrease	Correct
6	19.03	29.11	Decrease	Correct
7	12.15	19.03	Decrease	Correct
8	8.26	12.15	Decrease	Correct
9	5.88	8.26	Decrease	Correct
10	4.32	5.88	Decrease	Correct
11	3.99	4.32	Decrease	Correct
12	3.85	–	–	–
Accuracy				0.90(=9/10)

In Table 1, it is easy to see that the control *U*(*t*) is equal to the number of reported cases at the previous week *t*−1 as derived in (13). For each time step *t*∈ [ 2,11], the number of reported cases is compared to *U*(*t*) and if it is greater than *U*(*t*), then the prediction is “Increase” and if it is less than *U*(*t*), then the prediction is “Decrease”. In this case, the number of correct predictions is 9 and the total number of predictions is 10. Hence, the accuracy of the prediction is 9/10=0.90.

**Fig. 2:**
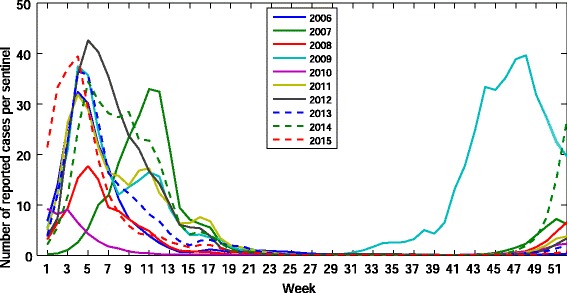
The time series of the actual number of reported cases per sentinel of influenza in Japan from 2006 to 2015

Next, we extend our prediction to all weeks from 2006 to 2015. The time series of the actual number of reported cases per sentinel in this period is illustrated in Fig. 2. In this case, the accuracy of our prediction for each year is listed in Table 2.

**Table 2:** The accuracy of our prediction for influenza in Japan from 2006 to 2015

Year	Number of correct	Number of incorrect	Accuracy
	predictions	predictions	
2006	44	6	0.88
2007	42	8	0.84
2008	45	5	0.90
2009	37	13	0.74
2010	35	15	0.70
2011	42	8	0.84
2012	38	12	0.76
2013	41	9	0.82
2014	41	9	0.82
2015	39	11	0.78
Total	404	96	0.81

As in the previous example, the prediction is not performed for the first week (*t*=1) and the last week (*t*=52). Therefore, the total number of predictions per year is 50. The accuracy of total predictions is 0.81 and relatively high.

### Comparison

Next we compare our prediction method with an alternative prediction method based on ARIMA (AutoRegressive Integrated Moving Average) models (see, for instance, Kane et al. [21]). To perform the alternative prediction method, we use the function “ARIMAProcess” implemented in Wolfram Mathematica 10.3. For each of the weeks in 2006-2015, we iteratively apply the actual data from each of the past 10 weeks (including the current week) to predict the number of reported cases at each of the next weeks. Note that we additionally need the data of the last 10 weeks in 2005 to perform the prediction at the beginning of 2006. If the predicted number for the next week is greater (less) than the reported number at the current week, then the prediction can be regarded as “Increase” (“Decrease”). As it is well known, ARIMA models *ARIMA*(*p*,*d*,*q*) can be characterized by the orders of the autoregressive part *p*, differencing *d* and moving-average process *q*. For different *p*,*d*,*q*∈[0,1], *p*+*d*+*q*>0, we obtain the prediction result as listed in Table 3.

**Table 3:** The accuracy of the alternative prediction based on *ARIMA*(*p*,*d*,*q*) models for influenza in Japan from 2006 to 2015 (total 500 predictions)

(*p*,*d*,*q*)	Number of correct	Number of incorrect	Accuracy
	predictions	predictions	
(0,0,1)	224	276	0.45
(0,1,0)	244	256	0.49
(1,0,0)	156	344	0.31
(0,1,1)	382	118	0.76
(1,0,1)	152	348	0.30
(1,1,0)	393	107	0.79
(1,1,1)	355	145	0.71

From Table 3, we see that (*p*,*d*,*q*)=(1,1,0) is the best choice in this case. Nonetheless, its accuracy is lower than that of our prediction method (0.79<0.81).

### Estimation of the number of reported cases

**Fig. 3:**
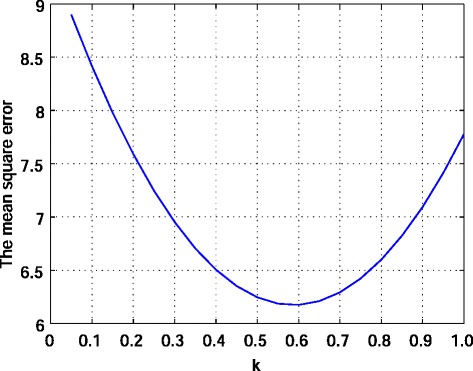
The mean square error between the actual number of reported cases per sentinel of influenza in Japan and the values estimated by our method (14) for 522 weeks from the first week of 2006 to the last week of 2015 with different fitting parameter *k*>0

**Fig. 4:**
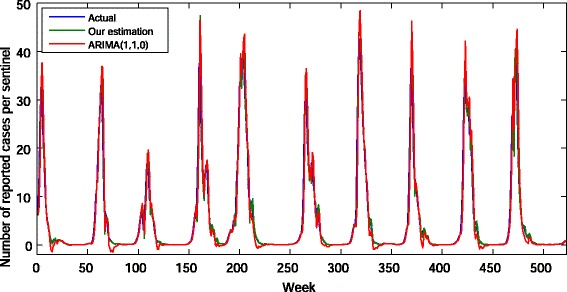
The time series of the actual number of reported cases per sentinel of influenza in Japan (*blue*) and the values estimated by our method (*green*) and *ARIMA*(1,1,0) (*red*) for 522 weeks from the first week of 2006 to the last week of 2015

Finally, we perform an estimation of the number of reported cases, which is consistent with our prediction method. Roughly, we assume that the increase or decrease of newly infected population at each week follows a linear law. That is,

14 $$ I(t+1,0) \approx I(t,0) + k \left(I(t,0)-U(t) \right),  $$

where *k*>0 is a fitting parameter. It is easy to see that (14) is consistent with our prediction method (that is, if *I*(*t*,0)>*U*(*t*), then *I*(*t*+1,0)>*I*(*t*,0) and if *I*(*t*,0)<*U*(*t*), then *I*(*t*+1,0)<*I*(*t*,0)). Using the actual data from 2005 to 2015, we perform an ongoing estimation from the first week of 2006 to the last week of 2015. To minimize the mean square error between the actual number of reported cases and our estimated values, *k* is chosen to be 0.6 (see Fig. 3). In this case, the mean square error of our estimation is 6.17457371, while that of the best-fitted ARIMA model *ARIMA*(1,1,0) is 9.469997. The estimation result is shown in Fig. 4. In Fig. 4, it is seen that although three curves take close values, some values estimated by *ARIMA*(1,1,0) are negative.

### Discussion

Although our prediction method only need the data from each of the past 2 weeks (including the current week), the accuracy of it (0.81) was higher than that of the best-fitted ARIMA model *ARIMA*(1,1,0) (0.79) which is based on the data from each of the past 10 weeks. Moreover, our estimation method (14) for the number of newly infected population was better than *ARIMA*(1,1,0) in the sense of mean square error. From these results, we conjecture that focusing only on the data in the current and previous weeks can lead to a good prediction for the spread of influenza in Japan.

## Conclusions

In this study, based on the backstepping method in engineering, we developed a new prediction method for the increase or decrease of newly infected population. Under the assumption that the period of infectiousness is same for all infected individuals (that is, the recovery rate is given by the Dirac delta function multiplied by a sufficiently large positive constant), the method was simplified to the comparison of the number of reported cases at the current and previous time steps. In spite of its simplicity, its accuracy was relatively high (0.81) for the spread of influenza in Japan from 2006 to 2015. Furthermore, the simple estimation method (14) based on the linear law was proposed and its accuracy was better in the sense of mean square error than that of the best-fitted ARIMA model *ARIMA*(1,1,0), which is based on the data from each of the past 10 weeks. From these results, we conjectured that focusing only on the data in the current and previous weeks can lead to a good prediction for the spread of influenza in Japan.

As future tasks, not limited to influenza in Japan, our prediction method would be applied to various infectious diseases in various countries. The assumption that the period of infectiousness is same for all infected individuals might have to be modified for each case. Our model (1) was based on the SIR epidemic model in which the newly infected individuals immediately have the infectiousness without latency. To consider more realistic situations, we might have to start the discussion from some different models such as the SEIR and SIRS epidemic models.
